# Ultraviolet index levels in Recife, Brazil: an ecological study to identify low-erythemal-risk sunlight exposure windows for infants

**DOI:** 10.1016/j.jped.2026.101618

**Published:** 2026-09-19

**Authors:** Marcelo Luis Urquia, José Roberto da Silva Junior, José Natal Figueiroa, João Guilherme Bezerra Alves

**Affiliations:** a College of Community and Global Health, Rady Faculty of Health Sciences, University of Manitoba, Winnipeg, Manitoba, Canada; b Instituto de Medicina Integral Prof. Fernando Figueira (IMIP), Recife, PE, Brazil

**Keywords:** Vitamin D, Sun exposure, Infants, Ultraviolet index, Recife, Northeast Brazil

## Abstract

**Objective:**

Current pediatric guidelines recommend avoiding direct sun exposure for infants under six months. However, these recommendations may offset other potential benefits of sunlight exposure. The authors aimed to examine the Ultraviolet Index (UVI) levels in Recife, Brazil, particularly during early-morning and late-afternoon periods.

**Methods:**

Ecological descriptive study using secondary data from the National Institute for Space Research (INPE - UV-Brazil Project). Hourly UVI averages at 6:00, 7:00, 8:00, and 9:00 a.m. and 3:00, 4:00, and 5:00 pm. for the year 2025 were collected, considering seasonal variations (rainy vs. dry season).

**Results:**

In Recife (8°S), the mean and maximum UVI remain ≤ 2.0 (low category) until 7:00 a.m. and after 04:00 pm. throughout the year according to WHO classification. During most of the rainy season (May-July), maximum UVI drops below 2.0 after 3:00 pm.

**Conclusion:**

In Recife, Northeast Brazil, maximum UVI values are in the WHO low category (≤ 2) until 7:00 a.m. and after 4:00 pm., indicating a low likelihood of acute erythema. However, these ecological data do not establish long-term carcinogenic safety in infants.

## Introduction

Vitamin D (25-hydroxyvitamin D) is a pleiotropic hormone essential for bone metabolism, calcium homeostasis, and immunoregulatory functions during childhood [1]. While dietary intake and oral supplementation contribute to serum levels, cutaneous synthesis triggered by Ultraviolet B (UVB) radiation (290–315 nm) remains the primary natural source for humans, accounting for approximately 80–90% of vitamin D in both healthy and unhealthy populations [[2], [3], [4]]. Recently, pediatric guidelines from the American Academy of Pediatrics (AAP) and the Brazilian Society of Pediatrics (SBP) have increasingly recommended that infants under six months of age avoid direct sunlight exposure due to the vulnerability of neonatal skin and the cumulative risk of skin cancer (melanoma and non-melanoma) in adulthood [5,6]. Consequently, routine oral supplementation of 400 IU/day is recommended for all infants, including those exclusively breastfed [6].

However, avoiding UVB sunlight exposure during the first six months of life may contribute to inadequate cutaneous vitamin D synthesis, increasing the risk of rickets, impaired bone mineralization, and hypocalcemia in infants who are not properly supplemented [5]. Beyond its role in calcium metabolism, UVB-induced vitamin D production may support immune maturation, potentially influencing susceptibility to respiratory infections and allergic diseases [7]. Moderate exposure to natural light may also contribute to circadian rhythm regulation, sleep–wake cycle organization, and overall well-being [8]. While direct and prolonged sun exposure is discouraged due to the sensitivity of infant skin, complete avoidance of UVB radiation may lead to unintended health consequences [[9], [10], [11]].

According to the World Health Organization (WHO), the Ultraviolet Index (UVI) is used to convey the risk of acute UV-related skin damage [12]. The UVI is, by construction, based on erythemally weighted irradiance using the CIE erythema action spectrum. A UVI ≤ 2 is classified as low and, in general, is unlikely to cause cutaneous erythema (sunburn) [12]. The Minimal Erythema Dose (MED) is the lowest UV radiation dose that produces perceptible erythema. However, erythema is not a surrogate for carcinogenic safety. DNA photodamage and immunologic effects may occur at sub-erythemal doses, and carcinogenesis reflects cumulative exposure over time [[13], [14], [15], [16], [17]]. Therefore, low-UVI periods can only be interpreted as periods of lower acute erythemal risk, not as periods without long-term oncogenic risk [18,19]. Nevertheless, the relative importance of sub-erythemal doses of sunlight in causing skin cancer in humans remains unknown [20]. In addition, whether early-morning and late-afternoon exposure provides sufficient UVB for meaningful vitamin D synthesis cannot be established from UVI alone, because vitamin-d-effective irradiance has a different action spectrum and is strongly influenced by solar zenith angle [20]. However, UVB radiation varies according to region, season, latitude, altitude, cloud cover, and air pollution [21]. Therefore, local studies are essential to characterize region-specific UV exposure [20]. This study aims to evaluate monthly UV radiation levels in Recife, Pernambuco, specifically focusing on the selected early-morning and late-afternoon hours.

## Methods

### Study design and setting

This is a descriptive, ecological study utilizing secondary time-series data on ultraviolet radiation in Recife, Brazil (Latitude: 8°04′03″ S; Longitude: 34°55′00″ W; Altitude: 4 m). Recife is characterized by a tropical monsoon climate with high solar radiation levels throughout the year, including the rainy season (typically April through July).

### Data sources

Data were retrieved from the National Institute for Space Research (INPE - UV-Brazil Project), Brazil’s primary meteorological monitoring agency. This project provides continuous monitoring of the UVI via ground-based sensors and satellite-derived estimates from the Ozone Monitoring Instrument (OMI) (https://satelite.cptec.inpe.br/uv/).

### Data collection and processing

The study analyzed UVI measurements at 6:00, 7:00, 8:00, 9:00 a.m., and 3:00, 4:00, and 5:00 p.m. to characterize early-morning and late-afternoon radiation levels. In this study, periods with UVI ≤ 2 were in the WHO low category. In Recife, sunrise and sunset times typically range from 5:00 to 5:30 a.m. and 5:15 to 5:45 p.m., respectively. Cloudy and rainy days were considered when the sky presented significant cloud cover or precipitation that attenuated incoming solar UV. These days were typically characterized by thick cloud layers, a high cloud fraction, or high cloud optical depth recorded by meteorological observations or satellite data. Further indicators included a reduction in measured UV irradiance compared with clear-sky model estimates for the same solar zenith angle, and persistent cloud cover for a significant portion of the measurement period (often evaluated using hourly observations or sky imagery). Under these conditions, clouds scatter and absorb part of the solar radiation, usually producing lower UVI values and greater temporal variability. Historical data were retrieved from daily INPE-UV-Brazil Project records, covering the period from January 1, 2025, to December 31, 2025.

### Analysis criteria (WHO standards)

UVI levels were classified according to the Global Solar UV Index (WHO/WMO/ICNIRP) as follows: low (0–2), moderate (3–5), and high to extreme (> 6)

### Statistical analysis

The data were analyzed using Stata software (version 12.0). Descriptive statistics, including means and 95% confidence intervals, were calculated for each month and specific hour of measurement. Mean annual UVI values at the selected hours were plotted before and after considering cloud cover. Ambient UVI represents erythemal-weighted irradiance, which does not necessarily reflect the actual individual exposure dose, since it depends on exposure duration, body surface area exposed, clothing, shade, posture, and surface reflectance. For reference, cumulative erythemal exposure can be expressed as Standard Erythemal Dose (SED); at a constant UVI of 2, 15 and 30 min correspond approximately to 0.45 and 0.90 SED, respectively. These values are environmental dose estimates and are not infant-specific safety thresholds. Day-level exceedance probabilities (e.g., percentage of individual days with UVI > 2 at 07:00 a.m.) cannot be reliably reconstructed from the monthly aggregated values presented here and require analysis of the original daily observations.

### Ethical aspects

This study utilized secondary, publicly available data retrieved from the National Institute for Space Research (INPE). As the data are anonymized, aggregated, and do not involve direct human participation or identifiable personal information, institutional review board (IRB) approval was not required, in accordance with the Brazilian National Health Council Resolution No 510/2016.

## Results

In 2025, Recife experienced no days with zero insolation, maintaining a nearly consistent 12-hour day length throughout the year due to its low latitude. The city averages 2250 to 2500 annual sunlight hours, with UVI measurements ranging from 0.1 to 7.0 during the selected early-morning and late-afternoon observation hours. Detailed monthly mean UVI values at selected hours and their respective confidence intervals are presented in Table 1. While these data assume clear-sky conditions, UVI values were significantly reduced when accounting for cloud cover and rainy days (Table 2). Mean annual radiation levels at the selected hours according to the season (dry versus rainy/cloudy) show UVI values consistently below 2.0 until 07:00 a.m. and after 4:00 p.m. (Figure 1). Minimum and maximum monthly UVI values assuming clear-sky conditions are presented in Table 3. Maximum UVI values only surpass 2.0 after 7:00 a.m. and drop below 2.0 after 4:00 p.m., except from May to July (rainy season), when maximum UVI values < 2.0 are also observed before 8:00 a.m. (July) and after 3:00 p.m. (May to July).

**Table 1:** Mean monthly Ultraviolet Index and 95% confidence intervals by hour, without considering cloud cover, Recife, 2025.

Month	6:00 a.m.	7:00 a.m.	8:00 a.m.	9:00 a.m.	3:00 p.m.	4:00 p.m.	5:00 p.m.
**January**	0.12 (0.10, 0.13)	1.19 (1.19, 1.20)	3.55 (3.47, 3.63)	6.94 (6.84, 7.04)	3.43 (3.31,3.54)	1.10(1.07, 1.13)	0.14 (0.12, 0.16)
**February**	0.10 (0.09, 0.11)	1.02 (1.01, 1.04)	3.34 (3.22, 3.45)	6.87 (6.85, 6.90)	3.82 (3.77, 3.88)	1.20 (1.18, 1.22)	0.18 (0.17, 0.20)
**March**	0.10 (0.10, 0.10)	1.00 (0.99, 1.02)	3.27 (3.23, 3.30)	6.95 (6.84, 7.06)	3.19 (3.11, 3.27)	0.95 (0.90, 1.00)	0.10 (0.10, 0.10)
**April**	0.10 (0.10, 0.10)	1.00 (1.00, 1.00)	3.13 (3.10, 3.16)	6.22 (6.13, 6.31)	2.36 (2.24, 2.49)	0.75 (0.72, 0.78)	0
**May**	0.10 (0.10, 0.10)	0.89 (0.80, 0.98)	2.88 (2.84, 2.91)	5.31 (5.18, 5.44)	1.85 (1.82, 1.87)	0.38 (0.36, 0.39)	0
**June**	0.07 (0.05, 0.09)	0.74 (0.71, 0.76)	2.10 (2.07, 2.13)	4.51 (4.41, 4.60)	1.81 (1.79, 1.82)	0.25 (0.23, 0.27)	0
**July**	0	0.69 (0.66, 0.73)	1.39 (1.23, 1.54)	4.53 (4.41, 4.66)	1.94 (1.91, 1.97)	0.41 (0.38, 0.45)	0
**August**	0.02 (0.01, 0.03)	0.92 (0.90, 0.95)	2.73 (2.60, 2.87)	5.53 (5.36, 5.70)	2.16 (2.12, 2.21)	0.77 (0.75, 0.80)	0
**September**	0.10 (0.10, 0.10)	1.31 (1.21, 1.40)	3.88 (3.76, 3.99)	7.16 (6.97, 7.35)	2.71 (2.51, 2.90)	0.79 (0.74, 0.83)	0
**October**	0.20 (0.18, 0.21)	1.96 (1.94, 1.98)	4.83 (4.57, 4.91)	8.14 (8.10, 8.18)	2.21 (2.19, 2.23)	0.59 (0.56, 0.62)	0.06 (0.04, 0.08)
**November**	0.22 (0.20, 0.24)	2.00 (2.00, 2.00)	4.92 (4.89, 4.95)	8.08 (8.04, 8.12)	2.23 (2.19, 2.26)	0.74 (0.71, 0.78)	0.10 (0.10, 0.10)
**December**	0.19 (0.18, 0.20)	1.70 (1.63, 1.78)	4.14 (4.07, 4.21)	7.38 (7.25, 7.50)	2.85 (2.78, 2.92)	0.96 (0.93, 0.99)	0.10 (0.10, 0.10)

**Table 2:** Mean monthly Ultraviolet Index and 95% confidence intervals by hour, considering cloud cover and rainy days, Recife, 2025.

Month	6:00 a.m.	7:00 a.m.	8:00 a.m.	9:00 a.m.	3:00 p.m.	4:00 p.m.	5:00 p.m.
**January**	0.07 (0.05, 0.09)	0.78 (0.65, 0.90)	2.29 (1.98, 2.60)	5.48 (4.88, 6.08)	2.82 (2.60, 3.04)	0.92 (0.82, 1.02)	0.10 (0.10, 0.10)
**February**	0	0.66 (0.57, 0.75)	1.75 (1.45, 2.04)	5.30 (4.73, 5.86)	3.28 (2.93, 3.62)	1.00 (0.88, 1.12)	0.11 (0.09, 0.12)
**March**	0	0.81 (0.73, 0.89)	2.53 (2.27, 2.79)	6.31 (6.03, 6.60)	2.61 (2.39, 2.84)	0.84 (0.78, 0.89)	0.01 (0.00, 0.03)
**April**	0	0.85 (0.79, 0.91)	2.80 (2.64, 2.95)	5.45 (5.17, 5.72)	1.88 (1.74, 2.01)	0.53 (0.45, 0.61)	0
**May**	0	0.62 (0.52, 0.71)	1.99 (1.66, 2.31)	4.25 (3.87, 4.63)	1.42 (1.28, 1.57)	0.25 (0.23, 0.27)	0
**June**	0	0.30 (0.25, 0.35)	1.56 (1.32, 1.80)	3.51 (3.17, 3.85)	1.42 (1.29, 1.56)	0.28 (0.24, 0.33)	0
**July**	0.01 (0.00, 0.02)	0.33(0.27, 0.38)	1.64 (1.49, 1.78)	3.47 (3.24, 3.70)	2.07 (1.89, 2.25)	0.22 (0.18, 0.25)	0
**August**	0	0.62 (0.54, 0.71)	2.25 (2.05, 2.46)	3.87 (3.38, 4.35)	1.78 (1.64, 1.91)	0.47 (0.36, 0.58)	0
**September**	0.03 (0.01, 0.04)	0.94 (0.87, 1.00)	3.36 (3.11, 3.60)	6.24 (5.81, 6.66)	1.89 (1.79, 1.98)	0.38 (0.32, 0.44)	0
**October**	0.10 (0.10, 0.10)	1.13 (1.08, 1.18)	3.67 (3.40, 3.93)	7.17 (6.78, 7.55)	2.21 (2.19, 2.23)	0.59 (0.56, 0.62)	0
**November**	0.09 (0.07, 0.10)	1.13 (1.04, 1.21)	3.62 (3.31, 3.94)	7.33 (6.89, 7.76)	2.02 (1.94, 2.09)	0.50 (0.44, 0.57)	0
**December**	0.06 (0.05, 0.08)	1.16 (1.04, 1.28)	3.26 (2.97, 3.54)	5.84 (5.35, 6.33)	2.33 (2.12, 2.54)	0.72 (0.63, 0.82)	0.10 (0.10, 0.10

**Figure 1:**
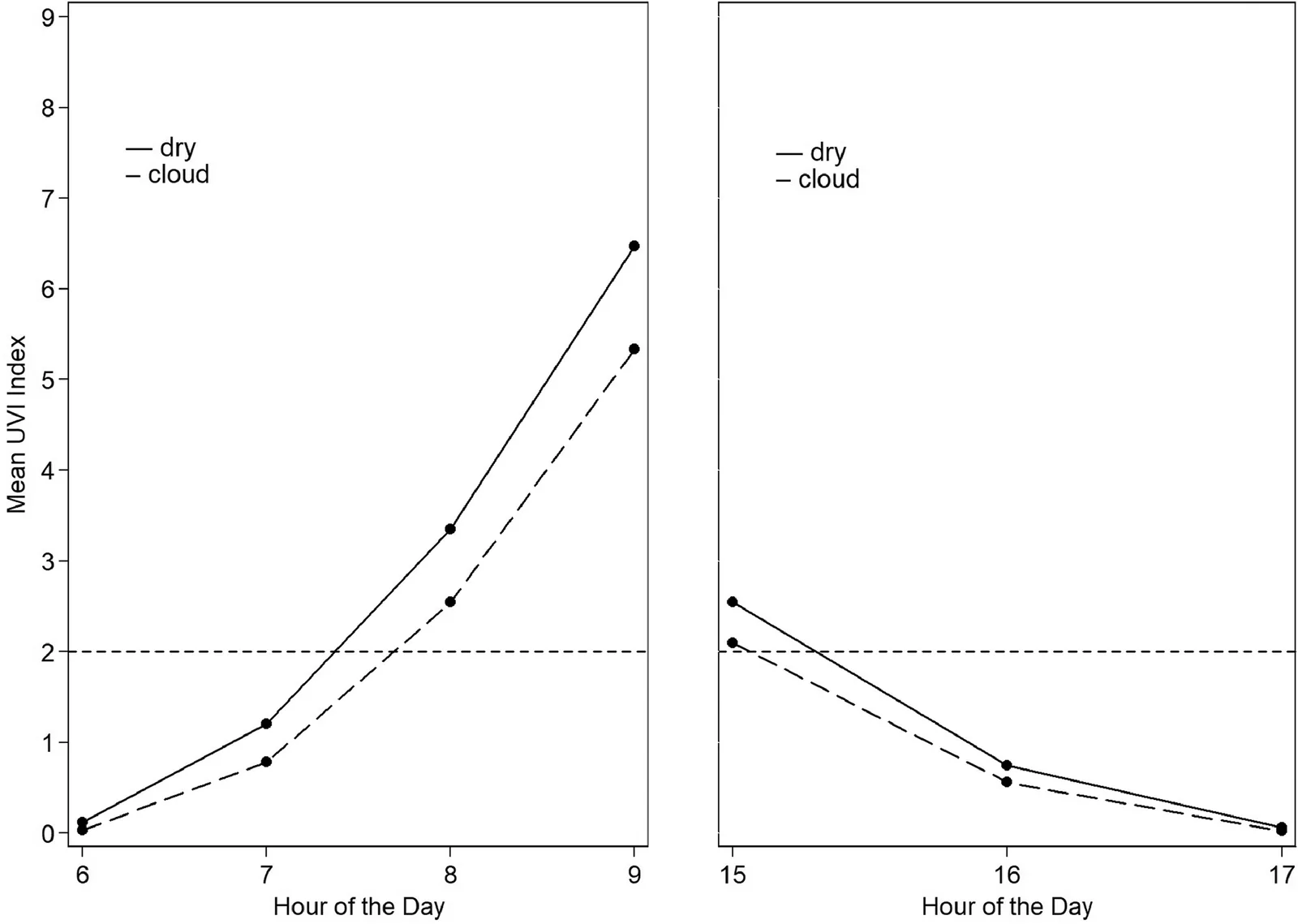
Mean annual Ultraviolet Index at selected hours according to the season (dry versus rainy/cloudy), Recife, Brazil, 2025.

**Table 3:** Minimum and Maximum monthly values of Ultraviolet Index, by hour, without considering cloud cover, Recife, 2025.

Month	6:00 a.m.	7:00 a.m.	8:00 a.m.	9:00 a.m.	3:00 p.m.	4:00 p.m.	5:00 p.m.
**January**	0.1, 0.2	1.0, 1.5	3.2, 4.1	6.8, 8.0	2.9, 3.9	1.0, 1.3	0.1, 0.2
**February**	0.1, 0.2	1.0, 1.1	3.2, 4.1	6.7, 7.0	3.2, 3.9	1.1, 1.2	0.1, 0.2
**March**	0.1, 0.1	0.9, 1.1	3.2, 3.5	6.8, 8.0	3.0, 3.8	0.3, 1.1	0.1, 0.1
**April**	0.1, 0.1	1.0, 1.0	3.0, 3.2	5.9, 6.8	2.0, 2.9	0.5, 0.9	0
**May**	0.1, 0.1	0.0, 1.0	2.7, 3.0	4.9, 5.9	1.8, 2.0	0.3, 0.4	0
**June**	0.0, 0.1	0.6, 0.8	2.0, 2.2	4.2, 4.9	1.7, 1.9	0.2, 0.3	0
**July**	0	0.6, 0.9	1.0, 2.0	4.2, 5.5	1.8, 2.0	0.2, 0.5	0
**August**	0.0, 0.1	0.8, 1.0	2.2, 3.1	5.0, 6.2	2.0, 2.5	0.6, 0.8	0
**September**	0.1, 0.1	1.0, 1.8	3.2, 4.4	6.2, 7.9	2.2, 3.6	0.2, 0.8	0
**October**	0.1, 0.2	1.9, 2.0	4.2, 5.0	7.9, 8.3	2.1, 2.4	0.5, 0.8	0.0, 0.1
**November**	0.2, 0.3	2.0, 2.0	4.8, 5.0	7.9, 8.2	2.1, 2.4	0.6, 0.9	0.1, 0.1
**December**	0.1, 0.2	1.3, 2.0	3.9, 4.5	7.0, 7.9	2.4, 3.1	0.9, 1.1	0.1, 0.1

## Discussion

This ecological time-series analysis demonstrates that in Recife, Brazil, the mean and maximum UVI remain below 2.0 before 7:00 a.m. and after 4:00 p.m. throughout the year, irrespective of the season. According to the Global Solar UVI classification proposed by the World Health Organization, UVI values between 0 and 2 are classified as low-risk and are unlikely to induce acute erythema in the general population [13]. From a photobiological standpoint, these findings identify reproducible daily windows with lower erythemally weighted irradiance. Importantly, the UVI is based on the CIE erythema action spectrum and therefore cannot by itself establish melanoma or non-melanoma skin cancer safety.

Skin cancer risk has been associated with UV exposure patterns that include intermittent intense exposure and sunburn (erythema). Intense UV can cause direct DNA damage, and early-life mutations may persist for decades, demonstrating a long latency period before cancer develops. Children have a thinner epidermis, lower melanin protection, immature DNA repair mechanisms, and a higher proportion of stem/progenitor cells, all of which can make them more vulnerable to UVR-induced skin damage, resulting in sunburns and increasing the risk of skin cancer later in life [22]. Concerns regarding infant skin immaturity are biologically valid [23]. However, emerging dermatologic data indicate that epidermal and barrier maturation extend throughout infancy rather than concluding abruptly at the age of six months [24]. This suggests that vulnerability is graded, not binary. Nevertheless, the long latency of melanoma does not imply that infant exposure is harmless; childhood UV exposure is an established melanoma risk factor [19], and early-life susceptibility should therefore be considered with particular caution.

Consistently, meta-analyses show that an increase in the number of sunburns at any age increases the risk of skin cancer [25,26]. However, absence of erythema does not imply absence of biological damage. Cyclobutane pyrimidine dimers and other DNA photoproducts can occur after sub-erythemal UV exposure, and sub-erythemal UV radiation may also induce immunosuppressive effects. The MED represents the lowest radiation dose capable of producing perceptible erythema at 24 h, but it should not be interpreted as a threshold for carcinogenic safety. Therefore, in the present study, UVI < 2.0 identifies periods of lower acute erythemal risk only and does not exclude cumulative long-term risk.

Cutaneous vitamin D synthesis remains an important physiological source of circulating 25(OH)D, accounting for approximately 80–90% under usual conditions [27,28]. While oral supplementation ensures adequacy, sunlight exposure may trigger other photobiological pathways, including nitric oxide mobilization and circadian effects. Recent perspectives have raised concerns that excessive sun avoidance may have broader public health implications [11].. However, current recommendations of the AAP and SBP appropriately emphasize infant photoprotection together with vitamin D supplementation. For infants, adequate vitamin D is essential for rickets prevention and normal bone mineralization. A potential limitation of the present rationale is that the identified low-UVI windows occur at high solar zenith angles, when the shorter UVB wavelengths most effective for previtamin-D3 synthesis are preferentially attenuated. Because the UVI is erythemally weighted and spectral irradiance was not available, meaningful vitamin D synthesis during these windows cannot be assumed from the present data and requires specific vitamin-D-weighted irradiance measurements or modeling.

This study utilizes high-precision, secondary data from the National Institute for Space Research (INPE), providing a robust longitudinal view of hourly radiation focused on clinically relevant time windows and including clear-sky and cloud-adjusted scenarios in a specific tropical context.

However, this study has several limitations that should be acknowledged. First, as an ecological study based on secondary environmental data, it did not measure serum 25-hydroxyvitamin D concentrations in infants; therefore, no direct association between the identified low-UVI exposure windows and vitamin D status can be established. Second, ambient UVI is not a measure of individual exposure dose, since it does not account for exposure duration, body surface area exposed, clothing, shade, posture, or reflectance. Third, individual skin phototypes (Fitzpatrick skin types), which substantially influence UV sensitivity, erythema thresholds, and vitamin D production, were not considered. Fourth, the UVI is based on the CIE erythema action spectrum; therefore, it is suitable for characterizing erythemal hazard but cannot establish a threshold for melanoma or keratinocyte-carcinoma safety. Fifth, vitamin-D-weighted irradiance could not be modeled from the available UVI data alone because spectral irradiance was not available. Sixth, the monthly aggregated data presented here do not permit reliable calculation of day-level exceedance probabilities, such as the percentage of days with UVI > 2 at 07:00 a.m. Although we reported maximum monthly UVI values that are useful to rule out the occurrence of UVI > 2 at certain months and times, these estimates represent the year 2025 and do not account for potential yearly variation. Finally, although the identified exposure windows presented low UVI values according to WHO criteria, this study did not evaluate the potential risk of hypo- or hyperthermia associated with environmental temperatures during early morning or late afternoon exposure. Therefore, recommendations for infant sun exposure should consider local ambient temperature, weather conditions, appropriate clothing, photoprotection guidance, and clinical judgment in addition to UVI values [28].

These limitations reinforce that the present ecological findings should be interpreted as hypothesis-generating and should not be used to define a clinically safe UV exposure threshold for infants.

In conclusion, Recife presents mean and maximum UV Index below 2.0 before 7:00 a.m. and after 4:00 p.m. throughout the year, identifying periods with a low likelihood of acute erythema according to the WHO UVI classification. Evidence is lacking for a specific threshold below which UV radiation can be considered carcinogenically safe in infants. Therefore, well-designed prospective studies incorporating personal UV dose, spectral/vitamin-d-weighted irradiance, serum 25(OH)D, skin phototype, and long-term outcomes are needed to clarify the dose-response relationship and biological effects at sub-erythemal levels in this age group.

## Funding sources

The study was not funded. MLU is supported by a Canada Research Chair in Applied Population Health (950–231324) of the Canadian Institutes of Health Research.

## Conflicts of interest

The authors declare no conflicts of interest.

## Data Availability

The data used for this study are publicly available at https://satelite.cptec.inpe.br/uv/. The dataset constructed for the study is available from the corresponding author upon reasonable request.
